# Oxaliplatin, fluorouracil and leucovorin for advanced biliary system adenocarcinomas: a prospective phase II trial

**DOI:** 10.1038/sj.bjc.6600543

**Published:** 2002-09-23

**Authors:** O Nehls, B Klump, H T Arkenau, H G Hass, A Greschniok, M Gregor, R Porschen

**Affiliations:** Department of Internal Medicine I, University Hospital, Otfried-Müller-Str. 10, 72076 Tübingen, Germany; Department of Pathology, University Hospital, Liebermeisterstr. 7, 72076 Tübingen, Germany; Clinic of Internal Medicine, Central Hospital Bremen Ost, Züricherstr. 40, 28325 Bremen, Germany

**Keywords:** biliary carcinoma, chemotherapy, 5-fluorouracil, oxaliplatin

## Abstract

We studied the activity of combined oxaliplatin and fluorouracil-leucovorin in 16 consecutive patients with advanced biliary tract adenocarcinomas. The disease control rate (responses and stable disease) was 56% (95% confidence interval, 29–84%) and the median overall survival time was 9.5 months (range 0.9–26.8+). Therefore, this regimen might be active in biliary adenocarcinomas with further evaluation necessary.

*British Journal of Cancer* (2002) **87**, 702–704. doi:10.1038/sj.bjc.6600543 www.bjcancer.com

© 2002 Cancer Research UK

## 

While surgery currently provides the only curative treatment option in tumours of the biliary system, the majority of patients is diagnosed with advanced stages of disease. Patients with stage IV (UICC) gallbladder carcinoma with distant metastases or with unresectable biliary carcinomas only treated by best supportive care, have a poor prognosis with a median survival time of less than 3 months (Pitt *et al*, 1997; Hejna *et al*, 1998).

However, in these tumour entities, palliative chemotherapy with single agents or combination therapies result in partial responses in about 10 to 20% of patients (Hejna *et al*, 1998). Moreover, innovative treatment modalities other than chemotherapy like photodynamic therapy have yielded preliminary beneficial results only in local tumour control but not for metastatic disease (Ortner *et al*, 1998). External beam radiation and brachytherapy have only demonstrated efficacy in individual patients (Hejna *et al*, 1998).

Based on the preclinical evidence that oxaliplatin combined with 5-FU-LV possesses remarkable synergistic cytotoxicity in various human malignancies (Raymond *et al*, 1997), this prospective phase II trial was initiated to investigate for the first time, to the best of our knowledge, the activity and safety profile of this combination therapy for advanced biliary system adenocarcinomas.

## PATIENTS AND METHODS

This prospective multicentre study enrolled 16 consecutive patients with histologically or cytologically confirmed adenocarcinomas of the gallbladder or the intrahepatic or extrahepatic biliary tract between November 1997 and May 2001. All patients had locally advanced, unresectable or metastatic disease and fulfilled the standard eligibility criteria (Raderer *et al*, 1999). The trial protocol was approved by the ethics committee of the University of Tübingen, and written consent was obtained from all patients before study entry.

The patients received the 2 weekly administered FOLFOX 3 regimen (André *et al*, 1998) including l-OHP 85 mg m^−2^ per 2 h on day 1 concurrent with LV 500 mg m^−2^ per 2 h, followed by continuous 5-FU infusion 1.5 to 2.0 g m^−2^ per 22 h on days 1–2. Objective tumour evaluation for response was performed at 9 week intervals according to WHO standard criteria (World Health Organization, 1979).

### Statistics

Overall survival (OS) was calculated from the initiation of chemotherapy until death. Time to progression (TTP) was determined by the interval between the start of chemotherapy and the date of objectively measured disease progression, or the occurence of death by any cause. Statistical analysis was performed using SPSS for Windows 10.0 (SPSS, Inc., Chicago, IL, USA). Median OS and TTP were estimated according to the Kaplan–Meier method (Kaplan & Maier, 1959).

## RESULTS

Patients characteristics are detailed in Table 1

**Table 1:**
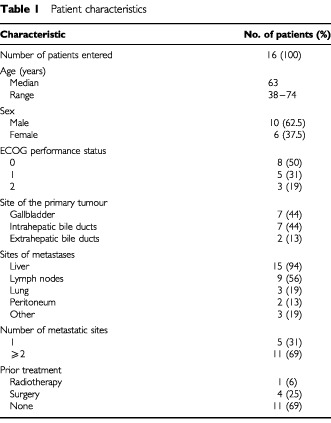
Patient characteristics

.

A total of 146 courses were administered (median 7, range 2 to 19). Thirty courses (21%) were delayed for at least 1 week due to unresolved haematologic (20 cycles) and non-haematologic (10 cycles) side effects. The median cumulative doses for oxaliplatin and 5-FU were 595 mg m^−2^ (range 170–1.615 mg m^−2^) and 12.350 mg m^−2^ (range 3.000–21.600 mg m^−2^) corresponding to 92.4 and 85.9% of the planned doses, respectively.

Fourteen of the 16 patients were evaluable for response by radiological imaging, including one patient who was withdrawn from the study after seven cycles due to an allergic reaction to oxaliplatin. In this patient response and survival were calculated on an intent-to-treat basis. Two other patients dying from rapid progressive disease after two or three treatment courses were included as non-responders.

Three patients (19%) achieved a partial response (95% CI, 0% to 41%) and six (37.5%) stable disease (95% CI, 11% to 64%). From initiation of chemotherapy, actuarial estimation of median TTP and median OS was 4.1 months (range 0.9 to 11.9) (95% CI, 0 to 11.3 months) and 9.5 months (range 0.9 to 26.8+) (95% CI, 0.4 to 18.6 months), respectively. At the end-point of this study three patients were still alive.

All 16 patients were evaluable for toxic effects on 146 courses. No toxic deaths occured on the study. Both WHO grade 4 adverse reactions (thrombocytopenia and non-febrile leukopenia) as well as grade 3 toxicities (thrombocytopenia, mucositis and infection) were experienced in less than 2% of cycles, respectively. The main grade 3 toxic effect was peripheral sensory neuropathy that was noted in three patients (4.8% of cycles). One patient experienced a febrile allergic reaction to oxaliplatin at the sixth treatment course. In the following cycle, therapy was given without oxaliplatin and was well tolerated, whereas re-introduction of oxaliplatin resulted in a febrile allergic reaction again. The patient was therefore withdrawn from the study.

## DISCUSSION

The search is on for novel options in the palliative treatment of advanced adenocarcinomas of the biliary system. Thus, in the present prospective phase II trial, we assessed the efficacy and toxicity profiles of a 2 weekly schedule of l-OHP combined with high-dose 5-FU-LV (FOLFOX-3 regimen) given on outpatient basis in this setting. Our results regarding efficacy, showing a disease control rate (responses and stable disease) of 56% and a median OS time of 9.5 months, are encouraging.

In previous studies for biliary tract tumours, including 5-FU-LV with or without etoposide (Glimelius *et al*, 1996), single-agent docetaxel (Papakostas *et al*, 2001) or weekly gemcitabine (1.200 mg m^−2^ and 1.000 mg m^−2^, respectively) (Raderer *et al*, 1999; Gebbia *et al*, 2001), median OS times have been reported in the range of 6.5 to 8 months, respectively. In two phase II trials, both including only small numbers of patients, gemcitabine plus 5-FU/LV and 2 weekly high-dose gemcitabine (2.200 mg m^−2^) yielded disease control rates of 59 and 66%, respectively (Gebbia *et al*, 2001; Penz *et al*, 2001).

Nevertheless, there is a need for larger study populations and randomized trials confirming these results. Of note, the underlying molecular mechanism of synergistic action of l-OHP plus 5-FU-LV combination therapy is completely different to the nucleoside analogue gemcitabine. Therefore, both regimens might be used for the treatment of advanced biliary carcinomas alternatively or as a sequential therapeutical approach, that might be addressed in future studies.

The toxic effects noted in our study are in accordance with those observed with the FOLFOX-3 regimen for the treatment of colorectal carcinoma (André *et al*, 1998) showing that this schedule is well tolerated.

In summary, this phase II trial suggests that oxaliplatin combined with 5-FU-LV is active and well tolerated in adenocarcinomas of the biliary system. Further studies, including an assessment in terms of quality of life, are required.

## References

[bib1] AndréTLouvetCRaymondETournigandCde GramontA1998 Bimonthly high-dose leucovorin, 5-fluorouracil infusion and oxaliplatin (FOLFOX 3) for metastatic colorectal cancer resistant to the same leucovorin and 5-fluorouracil regimen Ann Oncol 9 12511253 9862058 10.1023/a:1008475122124

[bib2] GebbiaVGiulianiFMaielloEColucciGVerderameFBorsellinoNMauceriGCarusoMTirritoMLValdesiM2001 Treatment of inoperable and/or metastatic biliary tree carcinomas with single agent gemcitabine or in combination with levofolinic acid and infusional fluorouracil: results of a multicenter phase II study J Clin Oncol 19 40894090 10.1200/JCO.2001.19.20.4089 11600613

[bib3] GlimeliusBHoffmanKSjödenPOJacobssonGSellströmHEhanderLKLinnéTSvenssonC1996 Chemotherapy improves survival and quality of life in advanced pancreatic and biliary cancer Ann Oncol 7 593600 8879373 10.1093/oxfordjournals.annonc.a010676

[bib4] HejnaMPruckmayerMRadererM1998 The role of chemotherapy and radiation in the management of biliary cancer: a review of the literature Eur J Cancer 34 977986 9849443 10.1016/s0959-8049(97)10166-6

[bib5] KaplanELMaierP1959 Non-Parametric estimation from incomplete oberservations J Am Stat Assoc 53 457481

[bib6] OrtnerMAEJLiebetruthJSchreiberSHanftMWruckUFuscoVMüllerJMHörtnageHLochsH1998 Photodynamic therapy in nonresectable cholangiocarcinoma Gastroenterology 114 536542 9496944 10.1016/s0016-5085(98)70537-2

[bib7] PapakostasPKouroussisChAndroulakisNSamelisGAravantinosGKalbakisKSarraESouglakosJKakolyrisSGeorgouliasV2001 First-line chemotherapy with docetaxel for unresectable or metastatic carcinoma of the biliary tract. A multicentre phase II stuy Eur J Cancer 37 18331838 11576836 10.1016/s0959-8049(01)00214-3

[bib8] PenzMKornekGVRadererMUlrich-PurHFiebigerWLenauerADepischDKraussGSchneeweissBScheithauerW2001 Phase II trial of two-weekly gemcitabine in patients with advanced biliary tract cancer Ann Oncol 12 183186 11300321 10.1023/a:1008352123009

[bib9] PittHAGrochowLBAbramsRA1997 Cancer of the biliary treeInCancer: Principles and Practice of Oncology5th (edn)DeVita VT, Hellman S, Rosenberg SA (eds)pp11141128Philadelphia, Pennsylvania/New York: Lippincott-Raven

[bib10] RadererMHejnaMHValencakJBKornekGVWeinländerGSBareckELenauerJBrodowiczTLangFScheithauerW1999 Two consecutive phase II studies of 5-fluorouracil/leucovorin/mitomycin C and of gemcitabine in patients with advanced biliary cancer Oncology 56 177180 10202270 10.1159/000011961

[bib11] RaymondEBuquet-FagotCDjelloulSMesterJCvitkovicEAllainPLouretCGespachC1997 Antitumoral activity of oxaliplatin in combination with 5-fluorouracil and the thymidylate synthase inhibitor AG337 in human colon, breast, and ovarian cancers Anticancer Drugs 8 876885 9402315 10.1097/00001813-199710000-00009

[bib12] World Health Organization1979 WHO Handbook for Reporting Results of Cancer Treatment (WHO Offset Publication No 48)Geneva, Switzerland, World Health Organization

